# Evaluation of the in vitro and in vivo effect of liposomal doxorubicin along with oncolytic Newcastle disease virus on 4T1 cell line: Animal preclinical research

**DOI:** 10.1002/vms3.1109

**Published:** 2023-03-15

**Authors:** Pooya Faranoush, Alireza Jahandideh, Reza Nekouian, Pejman Mortazavi

**Affiliations:** ^1^ Faculty of Specialized Veterinary Sciences, Science and Research Branch Islamic Azad University Tehran Iran; ^2^ Pediatric Growth and Development Research Center Institute of Endocrinology and Metabolism, Iran University of Medical Sciences Tehran Iran; ^3^ Nano Bio Electronic Devices Lab, Cancer Electronics Research Group, School of Electrical and Computer Engineering, College of Engineering University of Tehran Tehran Iran; ^4^ Department of Clinical Science, Faculty of Specialized Veterinary Sciences, Science and Research Branch Islamic Azad University Tehran Iran; ^5^ Department of Medical Biotechnology, School of Allied Medicine Iran University of Medical Sciences Tehran Iran; ^6^ Department of Pathology, Faculty of Specialized Veterinary Sciences, Science and Research Branch Islamic Azad University Tehran Iran

**Keywords:** breast cancer, liposomal doxorubicin, Newcastle disease virus, oncolytic virus, 4T1 cell line

## Abstract

**Background:**

Breast cancer is one of the most common malignancies in women, with one in 20 globally. Oncolytic viruses have recently been the first step in the biological treatment of cancer, either genetically engineered or naturally occurring. They increase specifically inside cancer cells and destroy them without damaging normal tissues or producing a host immune response against tumour cells or expressing transgenes. One of the most known members of this family is the Newcastle disease virus (NDV), a natural oncolytic virus that selectively induces apoptosis and DNA fragmentation in human cancer cells.

**Methods:**

This study performed biochemical and molecular investigations with variable doses of NDV (32, 64, 128 HAU) and liposomal doxorubicin (9 mg/kg) on mouse triple‐negative mammary carcinoma cell line 4T1 and BALB/c models tumours for the first time.

**Results:**

Real‐time quantitative PCR analysis in NDV‐treated animal tumours showed increased expression of P21, P27 and P53 genes and decreased expression of CD34, integrin Alpha 5, VEGF and VEGF‐R genes. Additional assessments in treated mouse models also showed that NDV increased ROS production, induced apoptosis, reduced tumour size and significantly improved prognosis, with no adverse effect on normal tissues.

**Conclusions:**

These findings all together might indicate that NDV in combination with chemotherapy drugs could improve prognosis in cancer patients although many more conditions should be considered.

## 1 INTRODUCTION

Breast cancer is one of the most common cancers, and its incidence and mortality rate is expected to increase yearly. Despite significant research advances, breast cancer remains a significant problem. The incidence of this invasive disease is rising alarmingly every year, indicating slow progress in prevention (Anastasiadi et al., 2017). High‐grade breast cancer management is complex due to treatment limitations and the tendency to resist standard treatments (O`Bryan & Mathis, 2018). In 2012, approximately 1.7 million people were diagnosed with breast cancer worldwide, and about half a million people died from this cancer (Ferlay et al., 2015). Significant prognostic factors include visceral, brain, and multiple metastases to other organs, which deteriorate the prognosis. In contrast, many cancers, such as bone cancer, and youngsters have a better prognosis (Mariotto et al., 2017). Treatment with oncolytic viruses is one of the most robust and most advanced methods in recent years. Oncolytic viruses are either genetically engineered or naturally occurring. They proliferate specifically inside cancer cells and destroy them without damaging normal tissues. Induce and initiate systemic antitumour and enhance the antitumour effect of the primary drug (Fukuhara et al., 2016; Msaouel et al., 2018). These viruses can directly kill tumour cells through various mechanisms, but they are insufficient to destroy tumour tissue completely and must be combined with other exogenous or other therapies (Wei et al., 2018). Newcastle disease virus is a single‐stranded, negative‐sense RNA virus with an envelope belonging to the genus Avulav of the family Paramyxoviridae. According to Newcastle studies, they are classified into lentogenic, mesogenic and velogenic groups. The velogenic strain results in 100% poultry mortality (Samour, 2014; Alexander, 2000). Although Newcastle disease causes severe infections in birds, it is nonpathogenic to humans. It can only cause conjunctivitis in humans, and no human‐to‐human transmission of the virus has been reported (Alexander, 2000). The LaSota Newcastle strain is the lentogenic type of virus and a natural oncolytic virus that selectively infects human tumour cells, binds to the malignant cell membrane, then enters the cytoplasm through endocytosis, causing apoptosis and DNA fragmentation in cancer cells (Al‐Ziaydi et al., 2020; Czeglédi et al., 2006; Al‐Shammari et al., 2016). Anthracyclines, especially doxorubicin, which has anticancer activity and significant toxicity, have long been isolated from the bacterium Streptomyces peucetius var, and they are the mainstay of cancer treatment. The first liposomal encapsulated anticancer drug to receive clinical confirmation against a wide range of tumours, including solid tumours, transplantable leukaemia and lymphoma, was doxorubicin. However, it has many side effects such as nausea, vomiting, alopecia, increased serum aminotransferase, acute liver injury and jaundice. Recent studies have focused on developing new liposomal formulations, A polyethylene glycol coating can wrap around the liposome to prevent it from phagocytising to form the liposomal doxorubicin, which is only 100 nm in diameter, that reduce the side effects of the drug, and because it is less toxic, higher doses of the drug can be prescribed, consequently advancing treatment (National Center for Biotechnology Information 2021; Tardi et al., 1996; Gabizon, 2001). Chemotherapy drugs have improved cancer treatment, but these drugs have many side effects, and recurrence of the disease has been seen in many cancers. So many efforts have been made to reduce the dose of chemotherapy drugs. The outcome is using chemotherapy drugs alongside oncolytic viruses to increase their function (Allen & Chonn, 1987; Ottolino‐Perry et al., 2010).

This study examines the effects of the Newcastle virus and liposomal doxorubicin drug together in the 4T1 cell line.

## 2 METHODS

### 2.1 Cell culture

Mouse triple‐negative mammary carcinoma cell line 4T1 were cultured in RPMI supplemented with 1% antibiotics (penicillin‐streptomycin, Gibco, 15140–172) and 10% foetal bovine serum (FBS, Gibco, 10270‐106). Cells were grown in a standard incubator at 37°C with 5% CO_2_ and 100% humidity. The media was changed every 2 days.

### 2.2 Animal tumour model

Female inbred BALB/c mouse models at the age of 3 weeks were housed at a temperature of 22−27^ο^C with a 12‐h light / dark cycle in a pathogen‐free isolation room. They were kept in this situation to adapt to the environment for a week. The Animal Ethics Committee approved all in vivo experiments in this study.

The mice were divided randomly into groups (5 mouse models per group). A total 1×10 (Wei et al., 2018) 4T1 cells/100 μL in the logarithmic growth phase were subcutaneously (sub‐Q) injected into the right flank region of the BALB/c mouse models. The size of tumour masses was determined every three days. Tumour volume was measured using the following equation (Faustino‐Rocha et al., 2013; Vassileva et al., 2008; Zandi et al., 2019).

$$\mathrm{Tumor}\mathrm{volume}=\left(\frac{4}{3}\right)\;\times \pi \times \left(\frac{\mathrm{width}}{2}\right)\times \left(\frac{\mathrm{Length}}{2}\right)\times \left(\frac{Depth}{2}\right)$$

### 2.3 LaSota strain of Newcastle disease virus

LaSota Newcastle is a lentogenic strain of the virus and is relatively stable in the standard environment (Czeglédi et al., 2006). For the proliferation of NDV, the particles were injected into the allantoic cavity of embryonated chicken eggs aged between 7–9 days. After being incubated for five days at 37°C, it was incubated at 4°C for 24 h to kill the foetus. In the last step, the NDV was harvested from the allantoic fluid and filtered through a 0.22 μm filter, and centrifuged (3000 rpm, 30 min, 4°C) have been used to purify it of debris for injected intraperitoneally in mouse models.

Haemagglutination assay (HA) test was performed to determine the proliferation of NDV (Santry et al., 2018). The haemagglutination units were shown per mL as HAU/mL.

### 2.4 Cell viability assay

The MTT (Methyl Thiazolyl Tetrazolium) assay was conducted to analyse the viability of the 4T1 cell line after infection with the NDV‐LaSota strain. The cells were cultured in 96‐well plates. Each well was filled with 10 (Ferlay et al., 2015) cells in a final volume of 200 μL and cultured in a standard cell culturing incubator (37°C, 5% CO_2_) for 24 h. When the total capacity of the well reached 70%, the 4T1 cell line was infected with LaSota NDV for various amounts of HAU, while the control wells remained untreated. The viability of the cells was analysed after one and 24‐h interactions of the NDV with 4T1 cell lines. After 1 day in each well, 3‐(4,5‐dimethylthiazol‐2‐yl)−2,5‐diphenyl tetrazolium bromide (MTT, Sigma‐Aldrich, Pro. No. M2128) solution (5 mg/mL) and 50 μL of RPMI (serum‐free cell media, Sigma, Pro. No. D5648) 50 μL was added and then incubated for 3 h in the standard cell culturing incubator (37°C, 5% CO_2_). 150 μL of dimethyl sulphoxide (DMSO, Sigma‐Aldrich, 334 Pro. No. D4540) was added in each well. Each evaluation was performed five times separately. For assess the IC50, the positive control cell population was compared to the outcomes and described as a percentage of the total cell population.

### 2.5 Cell cycle analysis

Cells were collected from the wells by trypsin and centrifuged (100g for 5 min at room temperature). The cell pellet was resuspended in phosphate‐buffered saline (PBS, Gibco, Pro. No. 70011–044), fixed with 70% ethanol, and stored at 4°C for 2 h. Then it was mixed with PBS with 100 μg/mL of RNaseA, 50 μg/mL of propidium iodide, and optionally 0.1% of Triton X‐100 (propidium iodide flow cytometry kit, Abcam, Pro. Co. ab139418). The container was wrapped in foil, stored at 4°C for 12 h and analysed by a flow cytometer (BD FACSCalibur, USA) and FlowJo (v10.5.3) software.

### 2.6 Apoptosis and necrosis detection

The 4T1 cell line was cultured on a 6‐well plate until it reached 70 to 80% of the total well capacity. Cells interacted with the IC50 dose of NDV in two groups (6 and 12 h). The treated cells were harvested by trypsin and centrifuged at room temperature for 5 min at 1200 rpm. The cell pellet was resuspended in phosphate‐buffered saline (PBS, Gibco, Pro. No. 70011–044). A Solution containing 100 μL of cells, 100 μL of incubation buffer with 2 μL of Annexin V (1 mg/mL, Annexin V‐FITC Apoptosis Staining, Abcam, Pro. Co. ab14085), and 2 μL of propidium iodide (1 mg/mL, PI, Abcam, Pro. Co. ab139418) was prepared. The cell pellet was resuspended in phosphate‐buffered saline and analysed by a flow cytometer (BD FACSCalibur, USA) and FlowJo (v10.5.3) software. Each experiment was assayed five times.

### 2.7 RNA extraction and real‐time PCR

**FIGURE 1:**
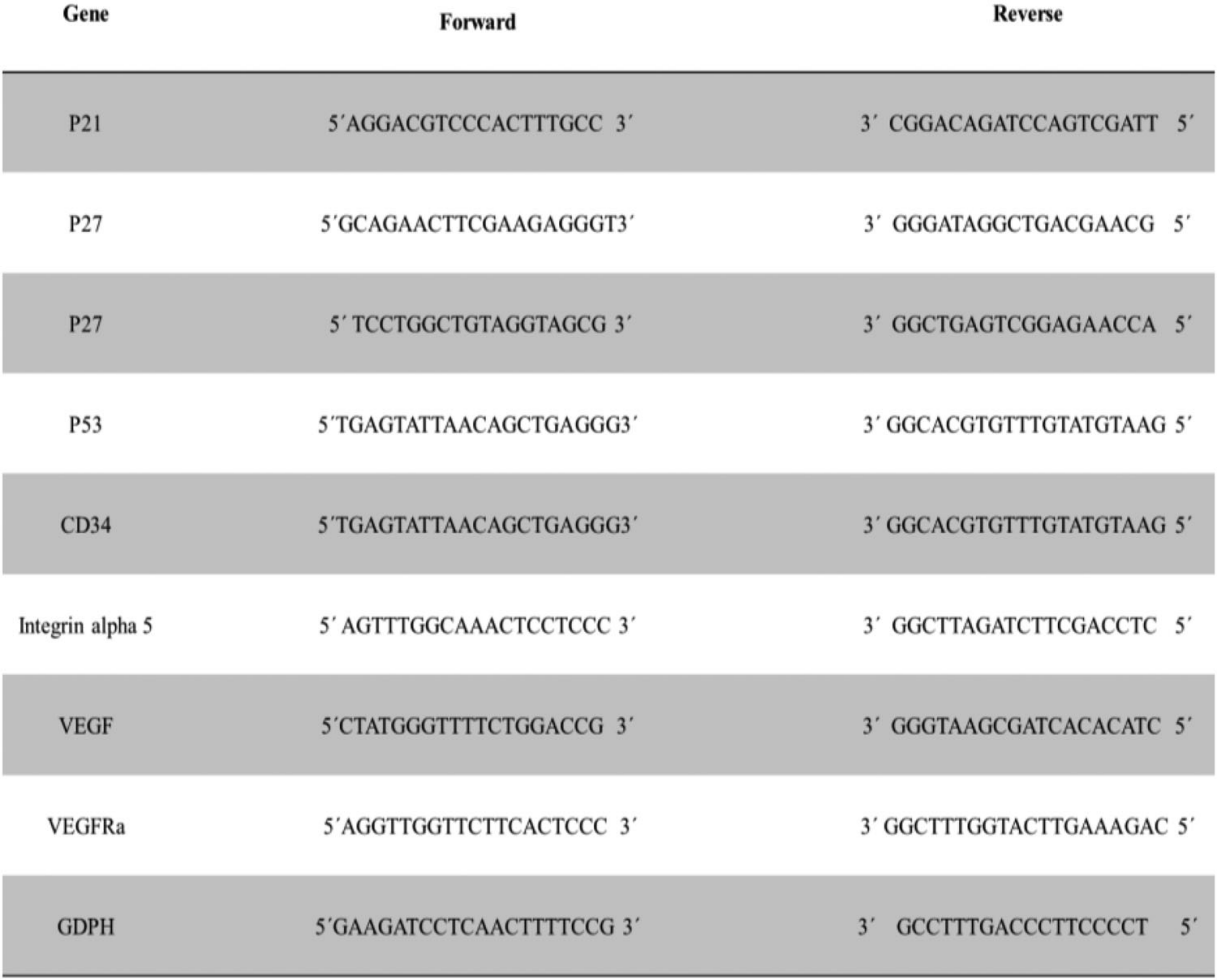
Real‐time RT‐PCR primers.

Total RNA was extracted from tumour tissue using the TRIzol reagent (Tiangen, Cat. No. 15596‐026, Beijing, China). 200 μL chloroform was added to the solution, shaken for 30 s, incubated at room temperature for 15 min, and centrifuged at 14,000 rpm at 4°C for 15 min. The supernatant was transferred to a new RNase‐free microtube with an equal volume of cold isopropanol and stored at minus 80°C for 30 min. The tubes were centrifuged at 14,000 rpm at 4°C for 10 min and mixed with 70% ethanol. Finally, the sample was centrifuged at 7000 rpm at 4°C for 10 min, then combined with Tris‐EDTA and kept at minus 80°C. The concentration of extracted RNA was measured using a UV spectrophotometer (NanoDrop One Microvolume UV‐Vis spectrophotometer, Thermofisher, USA). cDNA was synthesised using reverse‐transcribed RNA by a kit (QuantiNova SYBR Green PCR) following the manufacturer's procedure. The reverse transcription reaction was performed at 37°C for 1 h, and the inactivation of the reverse‐transcriptase condition was at 70°C for 5 min. The DNA polymerase activation was conducted at 95°C for 5 min, with 99 cycles of a two‐step PCR (95°C for 10 s and 55°C for 40 s). A dissociation curve analysis of P21, P27, P53, VEGF, VEGF‐A, integrinα5 and GAPDH showed a single peak. The SYBR Green real‐time RT‐PCR primers are reported in Supplementary Figure S1 (Thermofisher Scientific, USA). (Figure 1).

### 2.8 Evaluation of intracellular reactive oxygen species (ROS) generation by fluorescence microscopic methods

The DCFH diacetate form (DCFH‐DA) was used to measure the net intracellular generation levels of ROS. The 4T1 cell line was cultured on a 48‐well plate. After it reached 70%–80% of the total well capacity, it interacts cells with IC50 dose of NDV in two groups (6 and 12 h). DCFH‐DA was added to the well and incubated for 45 min. The wells were washed twice with PBS. Cells were imaged in the epifluorescence mode with a blue filter. All observations and control processes were perpetrated with a computer connected by IEEE1394.

### 2.9 Histological staining

Slides from tumours samples, spleen, liver, stomach, heart, brain, kidneys and lungs were collected for haematoxylin and eosin (H&E). The selected tissues were fixed in 10% neutral buffered formalin (NPF, fixative, Sigma‐Aldrich, HT501128) for 15 min. They were dunked successively in the 10% NPF for 2 h, ethanol (Sigma‐Aldrich, Pro. No. 1117270500) 70%, 80%, 90%, and 100% for 1 h, sequentially, xylene (Sigma‐ Aldrich, Pro. No. 534056) for 1 h, 60°C paraffin (Paraplast Plus, Sigma‐Aldrich, Pro. No. P368) for 2 h. Subsequently, a manual rotary microtome (Leica) was used to prepare sections with a diameter of 5 μm. Eventually, for morphological and histopathological examination with an optical microscope, haematoxylin and eosin (H&E) staining was used.

### 2.10 Immunohistochemical staining

A manual rotary microtome (Leica) was used to cut the embedded paraffin with 2 mm thick sections. After that, the sections were rehydrated in Coplin jars with xylene (Sigma‐Aldrich, Pro. No. 534056) for 4 min, 1:1 ratio of xylazine and 100% ethanol (Sigma‐Aldrich, Pro. No. 1117270500) for 4 min, then ratio of xylazine to ethanol 100% for 4 min, 95%, 70% and 50% each for 4 min, consecutively. Three per cent hydrogen peroxidase was used to prevent high nonspecific background staining. A series of mouse antihuman monoclonal antibodies (1:100) Ki67, P53, ER, PR, HER‐2 (all from Abcam, Pro. Co. ab279657, ab176243, ab16460, ab2765, ab16901) were used to the sections diluted in phosphate‐buffered saline (PBS) including 5% bovine serum albumin (BSA, Sigma‐Aldrich, pro. no. A3294) and 0.1% TWEEN‐20 (PBST, Sigma‐Aldrich, pro. no. 524653). The sections were incubated for 24 h at 4°C. Afterwards, they interacted with secondary antibodies (1:100), avidin‐biotin‐peroxidase (Abcam, Pro. Co. ab64212), diaminobenzidine (DAB, ScyTek Laboratories, ACK500) and mouse secondary antibody (Jackson Immuno Research Labs). In the last stage, haematoxylin (Richard‐Allen Scientific, Cat. No. 7211) was utilised as the counterstaining.

### 2.11 Chemotherapy regimens

Two groups of five mouse models with 4T1 tumours (with and without IC50 dose of NDV injection) were randomly allocated to various treatment groups on day 14 of tumour inoculation, were used chemotherapy regimens. The chemotherapy groups were treated with liposomal doxorubicin (sinadoxosome, liposomal) via an intraperitoneal bolus injection of 9 mg/kg/week for three successive weeks. Cardiotoxicity, skin toxicity, mucositis, weight loss, anaemia, allergic reactions, vomiting, alopecia and any other signs or symptoms of toxicity were evaluated (Ansari et al., 2017; Coleman et al., 2006). All mouse models were followed until relapse of tumours or death.

### 2.12 Data analysis and statistics

All data are described as standard deviation (SD). The normal allocation of each set was determined using a two‐way analysis of variance (two‐way ANOVA), and the datasets that did not have a normal distribution were normalised in SPSS software with a two‐step approach. The level of significance was set to *p* < 0.05.

To evaluate the cytotoxicity, and the apoptotic effect of LaSota strain on 4T1 cell line, GraphPad Prism version 8.4.0 and SPSS version 22.0 were used for the statistical significance analyses.

## 3 RESULTS

### 3.1 In vitro effects of Newcastle disease virus LaSota strain on 4T1 cell line

**FIGURE 2:**
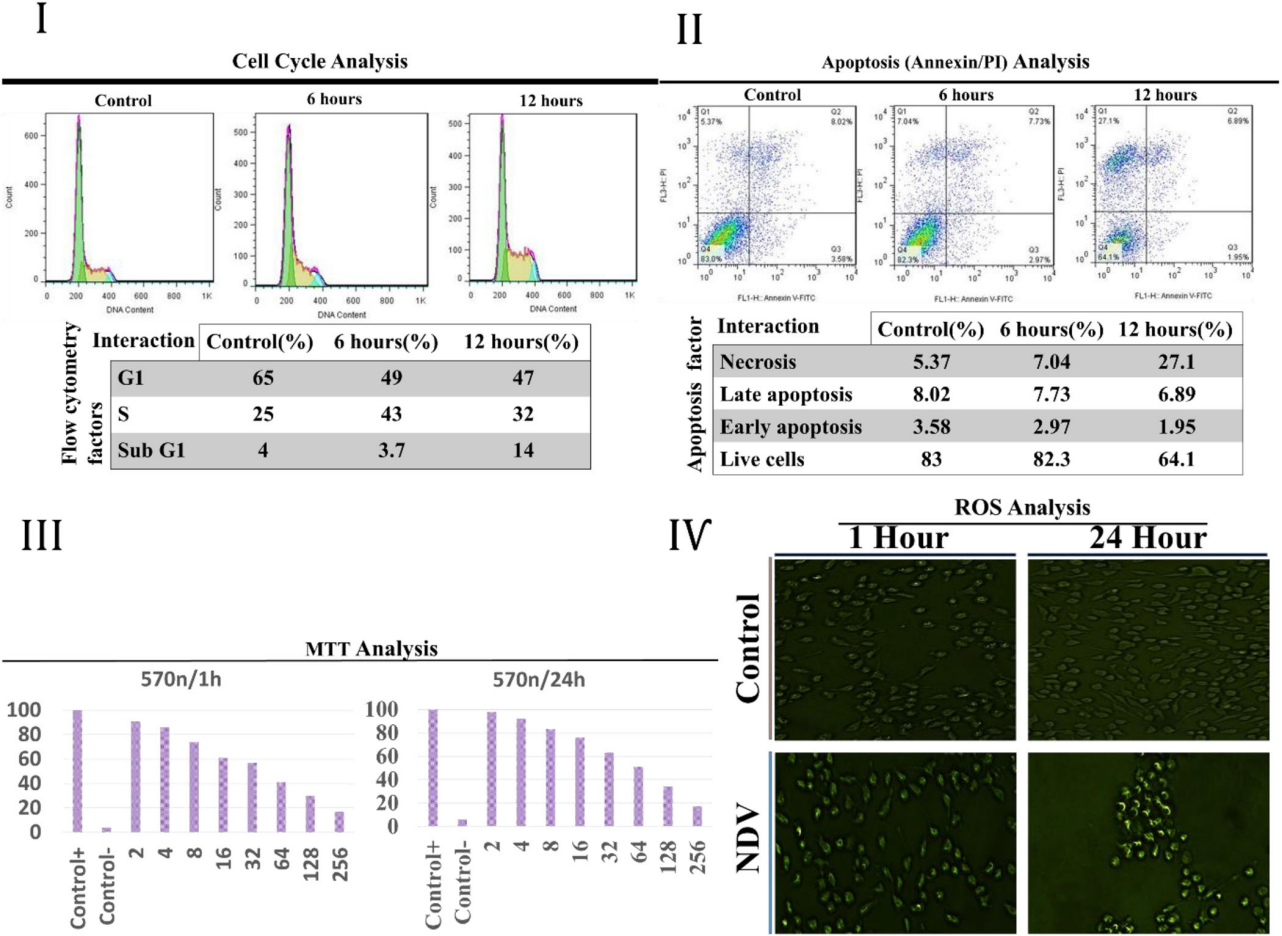
(I) The table summarises the flow cytometry‐based analysis of samples with two different interactions in the 4T1 cell line. The G1 stage represented a decreasing trend in the viability of the cells, while the Sub G1 and S stages showed an increasing trend. (II) Annexin/PI analysis was conducted on control and NDV samples to evaluate early and late apoptosis regarding the duration of the cell interaction. Increasing interaction time demonstrated an uptrend in the rate of apoptosis. All the data are displayed as mean ± SD. (III) MTT analysis showed that increasing the dose of NDV reduced the absorption in a 4T1 cell line. Data was gathered from 5 independent biological replicates of each test and analysed with an independent *t*‐test method. (IѴ) Fluorescence microscopic images related to the measurement of intracellular ROS by DCFH‐DA staining showed high DCF fluorescence in the NDV treatment after 24 h of interaction.

Initially, the effects of the Newcastle disease virus, LaSota strain on the 4T1 cell line with 6‐h and 12‐h interactions were examined by various methods such as flow cytometry to determine cell viability or evaluate the level of necrosis that showed a decreasing course in the G1 phase from 65% to 47% and an ascending course in phase S and Sub G1 (Figure 2I).

The highest titre of NDV‐LaSota strain for the MTT experiment to measure the effect of NDV on cell growth was 256 HAU. The IC50 titre was obtained 64 HAU by inducing 50% mortality of 4T1 cells.

The Annexin/PI test was used to evaluate the actual apoptosis rate in the 4T1 cell line after 6 and 12 h of exposure to NDV. The results of primary apoptosis depicted a downwards trend from 3.58% to 2.97% and 1.95% at 6 and 12 h, sequentially. The results of primary apoptosis depicted a downwards trend from 3.58% to 2.97% and 1.95% at 6 and 12 h, sequentially, as well as late apoptosis that decreased from 8.02% to 7.73% and 6.89% in the mentioned interactions. Live cell counts showed a reduction from 83% to 64% (Figure 2II).

MTT test was used to show the effect of NDV on cell growth. 8HA was the highest titre of NDV‐LaSota strain evaluated in the MTT test, and the IC50 titre was 64 HAU (Figure 2III).

A dose‐ and time‐dependent increase in the mean value of green fluorescence is observed. A significant in DCF (dichlorodihydrofluorescein) fluorescence was observed in 60 min of NDV 64 (HAU) treatment, which indicates an increase in ROS production in NDV‐treated cells were measured under a fluorescence microscope (Figure 2IV).

### 3.2 In vivo inhibition of mammary tumour growth by Newcastle disease virus LaSota strain in mouse models

A total of 1 × 10^6^/100 μL of 4T1 cancer cells (triple‐negative breast cancer mouse model cell line) were subcutaneously injected into the female BALB/c mouse models' right flank and followed up until the tumour volume reached ∼1000 mm^3^ to evaluate whether NDV inhibits malignant tumour growth in vivo. Afterwards, the mouse models were divided into seven groups. The NDV were administrated in three different doses, 32, 64 and 128 HAU, respectively. Another group was treated with NDV (64HAU) in conjunction with liposomal doxorubicin (3 mg/kg). The other group was only treated with liposomal doxorubicin (9 mg/kg). The last groups were considered as the placebo and control. All injections were performed seven days apart.

**FIGURE 3:**
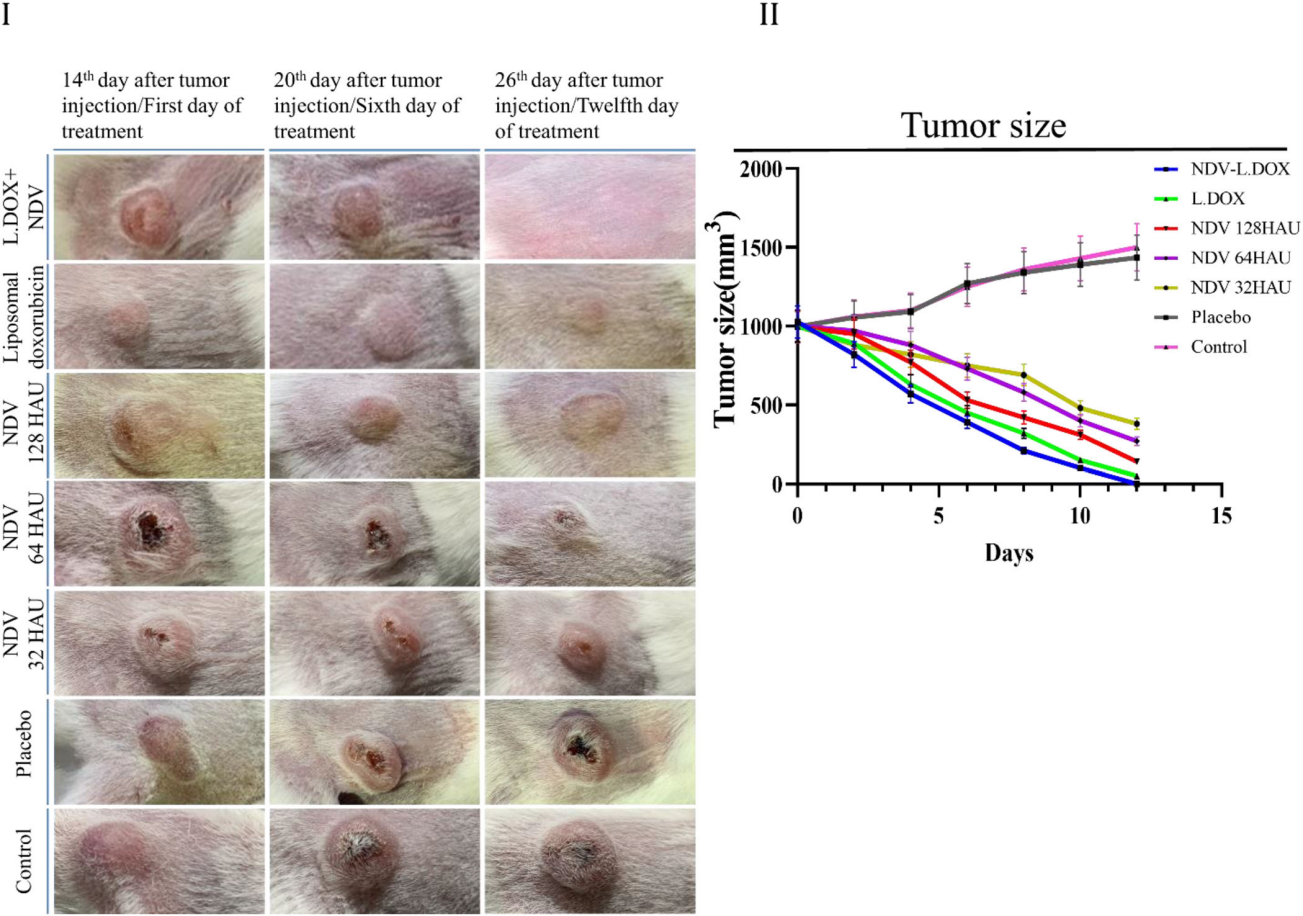
(I) Comparison of tumour sizes in each group showed that the treated mouse models significantly reduced their tumour size. (II) Tumour size alterations up to 15 days after the first day of treatment indicate that the tumour size decreased with an increasing drug dose.

**FIGURE 4:**
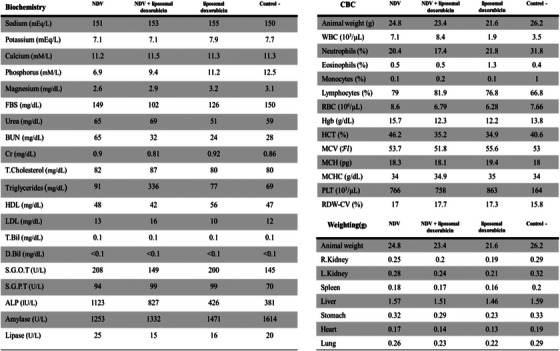
Comparison of blood characterisation and weight of mouse models and their organs in the groups compared to the control group demonstrated that blood factors and body weight in mouse models treated with NDV and liposomal doxorubicin were not significantly distinct from the control group.

Images taken from the treated mouse models showed an outstanding reduction in tumour size when the NDV and liposomal doxorubicin inject together. Tumour degradation in NDV‐treated mouse models proved a special relationship between tumour degradation rate and dose of NDV. So that after 21 days, a significant reduction in tumour size was observed even in low doses. After approximately 21 days of continuous injection, the mouse models treated with doxorubicin became tumour‐free (Figure 3I). However, in mouse models treated with IC50 dose of NDV and doxorubicin together, the tumour size reduction rate was 160–170 mm (Ferlay et al., 2015) per day, and no tumours were detected after about 10 days (Figure 3II). It was astonishing to figure out that the tumour size in mouse models treated with IC50 dose of NDV and doxorubicin together reduced by roughly a quarter in less than five days without affecting other organs such as the heart, skin, brain or kidneys. Examination of the body weight and organs of mouse models in the treated groups and control groups showed that the Newcastle virus did not cause a significant change in the weight of mouse models. Additional tests, such as complete blood count (CBC), serum electrolyte and chemistry, liver and kidney function test, amylase, and lipase, demonstrated no abnormalities in the typical mouse models treated by NDV compared to the control group (Figure 4). The long‐term effects of NDV were evaluated over 1 year. No complications were seen in NDV‐treated mouse models, and they became pregnant twice during this period. Also, the next generation had no complications and was able to reproduce.

### 3.3 Comparative histopathological examinations of treated and control tumourised mouse models

**FIGURE 5:**
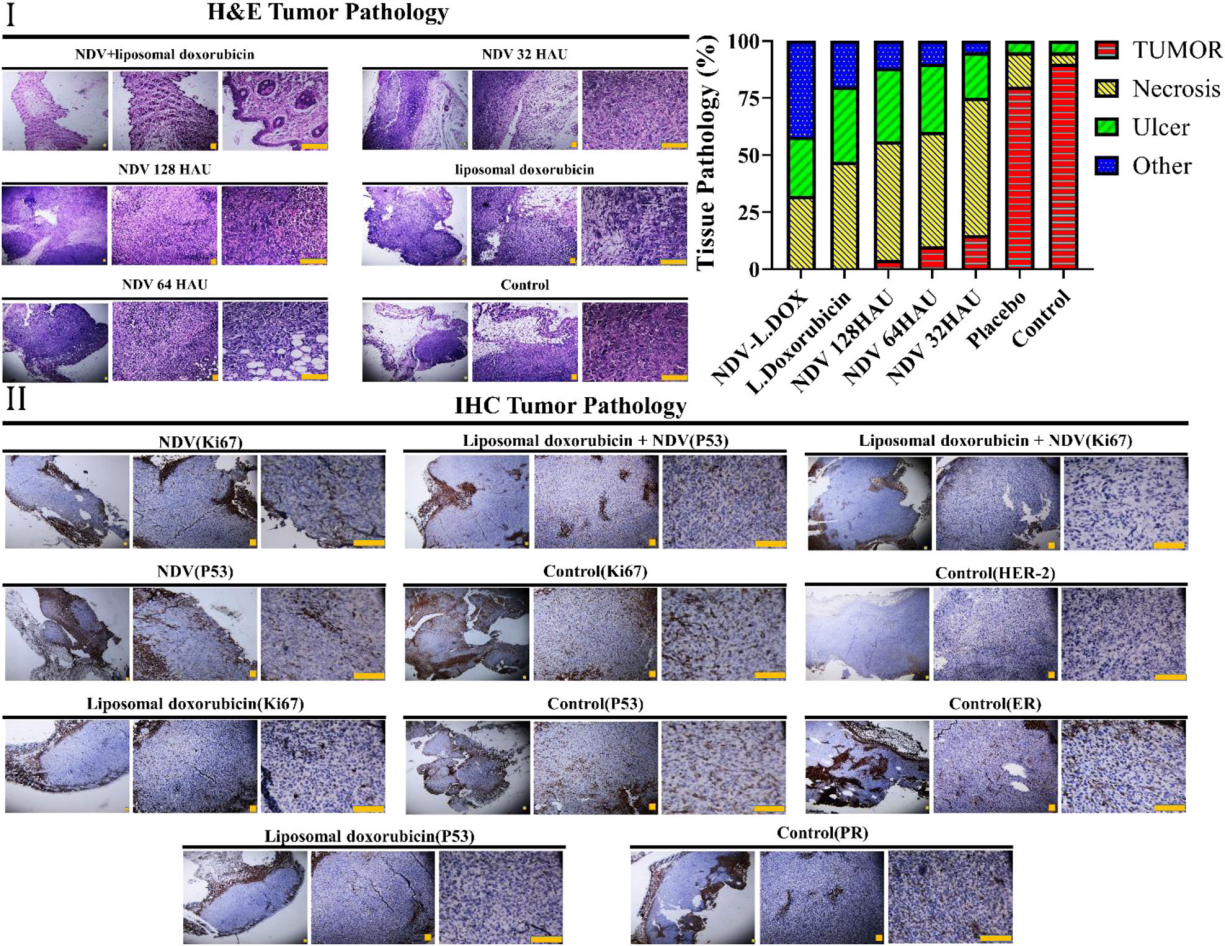
(I) The histopathological characteristics indicated tumoural tissue remains intact in the control group, whereas no tumour tissue was observed in the NDV+liposomal doxorubicin group. Each group includes five mouse models. The scale bars are set to 5 μm. (II) Immunohistochemistry analysis revealed downregulation and upregulation of Ki67 and P53 as proliferative and apoptotic markers, respectively and Overexpression of Ki67 in the control group showed high levels of proliferation. The scale bars are set to 5 μm.

**FIGURE 6:**
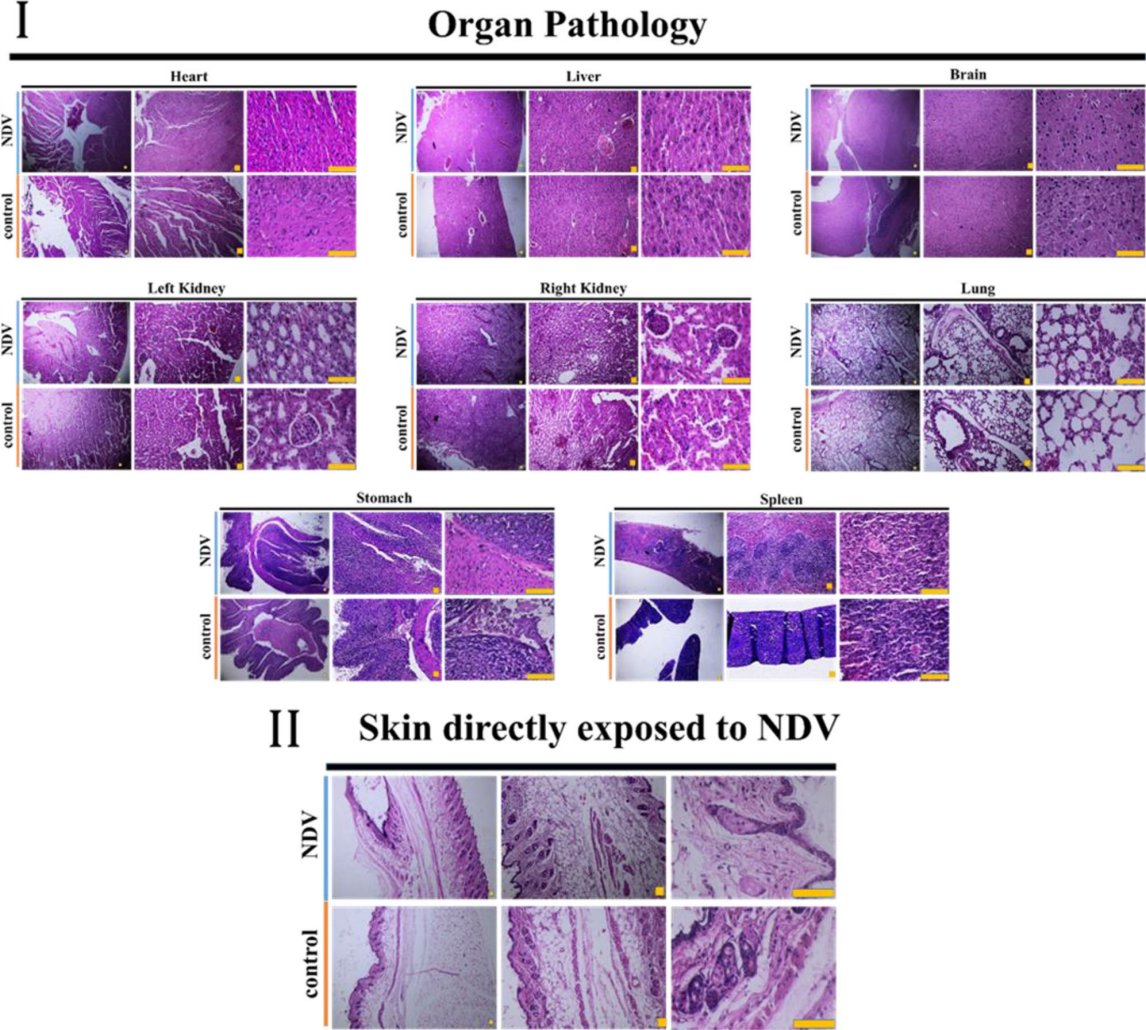
(I) Histological examination indicated that NDV eliminates tumour cells, in addition, it does not cause any damage to vital organs, including the liver, kidneys, spleen, lungs, stomach, brain, and heart. The scale bars are set to 5 μm. (II) Histopathological analysis of the sections directly exposed to NDV demonstrates no evidence of apoptosis or necrosis. Furthermore, it can be seen that cellular assembly and morphology stayed intact. The scale bars are set to 5 μm.

Highly condensed nuclei are called pyknotic nuclei (Elmore, 2007), which in the H&E image, it is used as a marker to detect cancer cells, and NDV activated internal apoptotic pathways of these cells. Histopathological analysis of NDV with doxorubicin‐treated mouse model tumours revealed a significant increase in apoptosis compared with the control and NDV or liposomal doxorubicin‐treated groups. Cytoplasmic degradation is predominant in Newcastle with liposomal doxorubicin‐treated malignant tissues; as the tissue matrix of the exposed tumours was not affected, the chance of necrosis was excluded (Figure 5I). To demonstrate selective apoptosis in the tumour mass used IHC staining with P53 and Ki67 as markers of apoptosis and proliferative, respectively (Ozer et al., 2012; Zandi et al., 2021) (Figure 5II). Also, H&E investigations of areas directly exposed to NDV as well as the body's vital organs, including the liver, kidneys, spleen, stomach, lungs, brain, and heart, were not demonstrated any pathological effects or any alternation, which indicates that the Newcastle virus will not negatively affect them (Figure 6I and II). Immunohistochemical staining revealed that NDV+liposomal doxorubicin‐treated tumours expressed higher P53 due to increased apoptotic and necrotic tumour cells and lower Ki67 expression as proliferative markers (Figure 5II).

**FIGURE 7:**
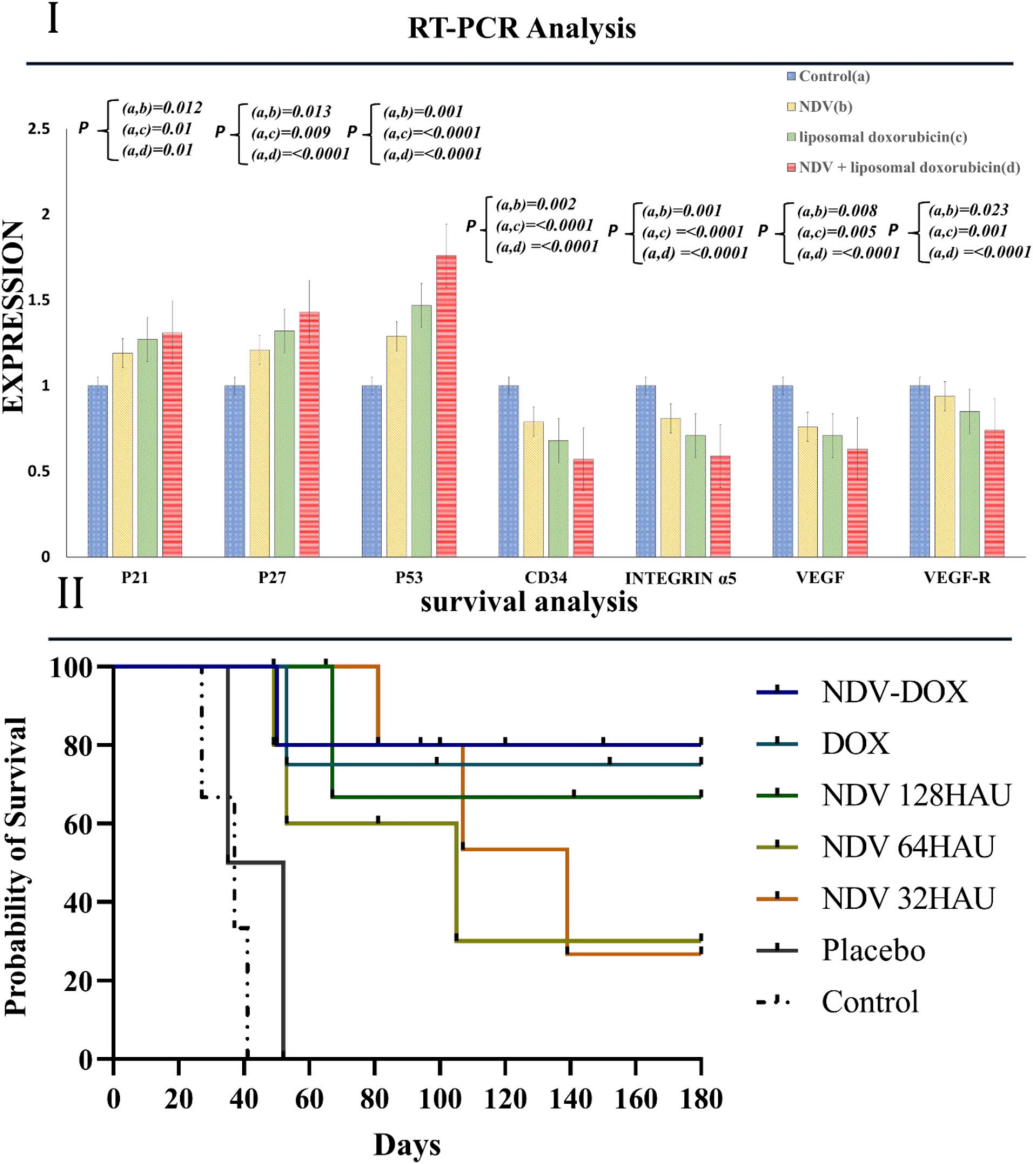
(I) Used real‐time reverse‐transcriptase polymerase chain reaction (RT‐PCR) to detect the expression level of P16, P21, P53, CD34, integrin α5, VEGF and VEGF‐R on day 26. NDV induced apoptosis in malignant cells via the overexpression of P21, P27 and P53 and downregulation of CD33, integrin α5, VEGF, and VEGF‐A in exposed groups. In this study, the GAPDH gene was used as a control. All the data are exhibited as means ± SD. (II) Kaplan–Meier survival curves of different mouse models in each group. There is no distinction between the control and placebo groups' survival curves (log‐rank test). NDV‐liposomal doxorubicin group had the most survival rate. Survival rates depended on the dose of NDV and decreased in the lower dose of NDV.

P21, P16 and P53 are components of the apoptosis pathway, which were significantly upregulated just in NDV‐treated tumours in quantitative real‐time polymerase chain reaction (RT‐PCR) analysis. Furthermore, spreading and adhesion transcriptomes including integrin α−5, vascular endothelial growth factor (VEGF), vascular endothelial growth factor receptor (VEGF‐R), and CD34 genes were downregulated in treated cohorts (Figure 7I).

### 3.4 survival analysis

All mouse models died within roughly 50 days in the control and placebo groups. The Kaplan–Meier survival curves for the NDV and NDV‐doxorubicin group demonstrated significantly higher survival rates than the control group, and they survived after 180 days. In addition, pathology analysis proved that tissues were treated with NDV‐liposomal doxorubicin had the least ulcer and necrosis (Figure 7II).

## 4 DISCUSSION

Oncolytic viruses are an essential therapeutic protocol against cancer. Newcastle disease virus is a natural oncolytic virus that induces adaptive immunity against tumours by selectively affecting the cancerous cells without harming the normal or untransformed ones (Wei et al., 2018; Schwaiger et al., 2017; Schirrmacher, 2015). In the study designed by Keshelava et al. (2009), it was found that NDV can play a crucial role in the treatment of cancer. Moreover, Hassan et al. (2020) studies in 2020 reported that the LaSota strain of NDV had an oncolytic activity against breast cancer cell lines, and mouse mammary adenocarcinoma. In this study, the effect of the Newcastle disease virus along with liposomal doxorubicin was investigated to prevent the side effects of chemotherapy drugs. An Annexin V/PI staining and ROS measurements were designed to initially investigate the oncolytic activity's mechanism on breast tumour cells. The NDV‐LaSota strain was found to induce effective oncolytic activity against 4T1 cells in a dose‐dependent manner. The MTT assay was performed with various concentrations of NDV as part of assessing and determining the oncolytic titre of NDV for cellular integrity and metabolism. In the *Cyperus rotundus* study, the NDV remarkably reduced the viability of cancer cells (AL‐Hilli et al., 2010). The investigation of the effect of NDV on human fibrosarcoma and fibroblast cells conducted by Krishnamurthy et al. (2006) determined that NDV can eliminate human fibrosarcoma cells quickly; also, in human skin fibroblasts as control cells, it had no effect. NDV replication is independent of DNA replication in the host cell, and it can inhibit cancer cells by cytolysis or viral replication, which induces apoptosis and breaks the therapy resistance of tumour cells (Schirrmacher, 2015; Elankumaran et al., 2006). Newcastle virus induces apoptosis in cancer cells by activating the death receptor (external) and mitochondrial (intrinsic) pathways. The drug combination is the most extensive method in treating cancer, and the main ambition is to reduce the drug dose and its side effect (Chou, 2010). Rajmani's investigations reported that NDV HN protein induces apoptosis in the human cervical cancer cell line by activating the internal SAPK/JNK pathway (Shim, 2015). Also, Ghrici demonstrated that the NDV activates mitochondrial transport pores and caspase‐8 and induces apoptosis in MCF‐7 human breast cancer cells (Ghrici et al., 2013). According to Gonin‐Giraud et al.’s and Al‐Ziaydi's studies, interference with the glycolysis cycle can be a valuable antitumour strategy. When apoptosis induces in cancer cells, they increase their glycolysis rate to meet the energy needed for survival. NDV can inhibit glycolysis. After that, lactate production is completely blocked, and intracellular ATP concentrations are abruptly reduced (Gonin‐Giraud et al., 2002; Al‐Ziaydi et al., 2020). Mozaffari Nejad et al. (2020) found that ROS levels in NDV‐treated cells increased. At the high ROS levels, apoptotic pathways are enhanced, and it is the main component of oxidative stress and contributes to cell death by inducing autophagy in the cancer cells (Scherz‐Shouval & Elazar, 2007; Levine & Klionsky, 2004). Moreover, a study designed by Obaid et al. (2022) demonstrated ROS induction by NDV as an antitumour mechanism. Our study showed increasing in ROS levels similar to the previous study.

NDV is safe for human cells and, in combination with other modalities of cancer treatment, can increase the therapeutic effect, improving patients’ survival and reducing the side effect of chemotherapy drugs with better quality of life.

## 5 CONCLUSION

In outline, we found an innate ability of NDV to induce selective apoptosis in cancer cells and annihilate malignant tumours in animal models with clinically validated outcomes. NDV was used to treat the tumours of female mouse models. The Newcastle virus in humans has very few side effects compared to chemotherapy. These side effects are temporary and disappear in 1 to 2 days. Histopathological analyses and cellular/molecular examinations demonstrated activation of apoptosis and suppression of proliferative markers in treated malignant cell lines and mouse model tumours.

Due to the many side effects of chemotherapy drugs, this method can be used as an adjunctive therapy to decrease side effects, increase the antitumour efficacy, and offer a novel insight into using oncolytic viruses, especially Newcastle disease virus, in cancer treatment.

## AUTHOR CONTRIBUTIONS

Pooya Faranoush: conceptualization; data curation; formal analysis; funding acquisition; investigation; methodology; project administration; resources; software; supervision; validation; visualization; writing‐original draft; writing‐review & editing. Alireza Jahandideh: validation; writing‐review & editing. Reza Nekouian: investigation; methodology; validation; writing‐review & editing. Pejman Mortazavi: validation. All authors provided critical review and editing to this manuscript. In addition, all authors have read and approved the final version of this manuscript.

## CONFLICT OF INTEREST STATEMENT

The authors declare no conflicts of interest.

## CONSENT FOR PUBLICATION

All authors have read and approved the final version of this manuscript for publication.

## FUNDING

The authors received no financial support for the research, authorship and/or publication of this article.

### ETHICS STATEMENT

The ethical committee of the Islamic Azad University, Science and Research Branch authorised the study, and all the examinations were performed with the ethical code of IR.IAU.SRB.REC.1400.329.

### PEER REVIEW

The peer review history for this article is available at https://publons.com/publon/10.1002/vms3.1109.

## Data Availability

The data that support the finding of this study are available from the corresponding author on request.
